# Classic Type of Epithelioid Sarcoma of the Distal Upper Extremity: Clinical and Oncological Characteristics

**DOI:** 10.1177/15589447221075745

**Published:** 2022-02-20

**Authors:** Farhad Farzaliyev, Hans-Ulrich Steinau, Andrej Ring, Paula Beck, Jendrik Hardes, Arne Streitbürger, Lars Erik Podleska

**Affiliations:** 1 Department of Trauma, Hand and Reconstructive Surgery/Division Plastic and Reconstructive Surgery, University Hospital Essen, Germany; 2 Department of Tumor Orthopedics and Sarcoma Surgery, University Hospital Essen, Germany; 3 Department of Plastic and Reconstructive Surgery, St. Rochus-Hospital, Castrop-Rauxel, Germany; 4 Ruhr-University Bochum, Germany

**Keywords:** tumor, diagnosis, surgery, specialty, wrist, anatomy, hand, anatomy, outcomes, research & health outcomes

## Abstract

**Background:**

The classic type of epithelioid sarcoma (ES) is a rare, aggressive soft tissue neoplasm that most commonly affects the distal upper extremities of young patients. This study aimed to assess clinical features and provide a long-term report of the oncological outcome.

**Methods:**

We retrospectively analyzed our clinical database for patients with ES of the distal upper extremities.

**Results:**

Twenty-three patients with ES of the distal upper extremity were treated surgically between January 1990 and August 2018. ES affected most commonly the palmar side of young patients. The most common site affected by a sarcoma was the wrist in 47.8% of cases, followed by metacarpals and fingers with 34.8% and 17.4%, respectively. Most of the patients were treated according to the protocols of interdisciplinary tumor boards with multimodal therapy. A local recurrence was observed in 7 patients (30.4%). The 5 - and 10-year recurrence-free survival was 80.4% (95% confidence interval [CI]: 68.6-76.8) and 60.9% (95% CI: 53.5-68.3), respectively. The 5- and 10-years disease-specific survival was 89.9% (95% CI: 87-92.8) and 61.9% (95% CI: 56.5-67.3), respectively. Five patients (21.7%) had metastasis in regional lymph nodes.

**Conclusion:**

The classic type of ES represents a group of high-grade sarcomas, which affect the dominantly distal upper extremity. Specific clinical, diagnostic, and oncological characteristics make it difficult to diagnose and therapy. Wide tumor resection as a part of multimodal therapy remains a more viable and common treatment option for patients with ES on distal extremities. High rates of lymph node metastasis are typical for ES.

## Introduction

The classic type of epithelioid sarcoma (ES) is a rare, aggressive soft tissue neoplasm most commonly affecting the distal upper extremities of young patients. It is composed of multinodular arrays of cells with only mild atypia and geographic necrosis. This tumor characteristically consists of a distinctive granuloma-like pattern of epithelioid cells of uncertain but multidirectional, predominantly epithelial differentiation, without a regular cellular counterpart. For comparison, the proximal variant has a predilection for proximal limbs and trunk of slightly older adults and comprises sheets of giant, more pleomorphic cells.^1-3^

Enzinger first described ES as a nodular or multinodular sarcoma that grows along fascia, tendons, and nerves.^4^ Infrequently, ES has its origin in the subcutis or dermis with possible ulceration of the overlying skin.^5^ It can easily be misinterpreted as a squamous cell carcinoma or other benign lesions such as Dupuytren's disease, rheumatoid nodule, granuloma annulare, chronic palmar ulcer, among others.^6-8^ Immunohistochemical stains help differentiate ES from other benign and malignant lesions. Moreover, it highlights the diversity of immunohistochemical findings demonstrated by ES, which confirms its differentiating capabilities. It does not describe its histogenetic but distinguishes it from other soft tissue sarcomas.^9^

Currently, limited data are available regarding the clinical features and management of ES of the distal upper extremity. Most of these data indicate differences in the course of this disease in comparison to other high-grade sarcomas.^10-12^ The main goal of the current study was to review clinical features and provide a long-term report of the oncological outcome.

## Materials and Methods

The data of 23 patients with ES of the distal upper extremity treated surgically between January 1990 and August 2018 were retrospectively analyzed. The study has been approved by the local ethics committee.

The diagnosis was made according to the clinical, radiological, and histological findings. For the patients from earlier, 90x computed tomography (CT)-scans of distal upper extremity; for other patients, magnetic resonance imaging (MRI) with contrast was performed to visualized detailed anatomic information for surgical planning. Due to the extensive growth of the tumor along the fascia, tendon, and nerves, tumor size was defined as the segment between the 2 most distant points. Chest CT, involving the Axilla region, was done to rule out distant lung and lymph node metastasis. An incisional biopsy was performed for previously nonbiopsied tumors or recurrences. Our pathologists performed secondary histological examinations for external biopsied sarcomas.

Multimodality treatments, including hyperthermic isolated limb perfusion with tumor necrosis factor (TNF)-alpha and Melphalan (TM-ILP), surgery, chemotherapy, and radiotherapy based on protocols of appropriate for the time of diagnosis, were appointed for these patients by interdisciplinary tumor boards. Radiotherapy was given to patients considered to be at risk of disease recurrence to improve local control rate.

At our centers TM-ILP has been available since early 2000s. Vascular access was gained by surgically placing the perfusion catheters into vessels under general anesthesia. Then 1 mg TNF-alpha (Beromun, Boehringer-Ingelheim, Ingelheim, Germany) and adapted doses of Melphalan were injected. Hyperthermic treatment was carried out at 39°C. Pathologists assessed response to TM-ILP by the amount of tumor necrosis. The disappearance of known disease was considered as complete response, presence up to 50% of the tumor cells in the specimen as partial response, and more than 50% as no response.^13^

The goal of surgery was to achieve negative surgical histopathological margins where possible in the setting of a limb-salvage therapy. Depending on the size of the tumor, localization on the distal upper extremity, relationship to the critical structures of this organ, and previous therapies, finger/ray amputation or extended resection including complex reconstruction, or limb amputation was performed.^14,15^ In case of positive resection margins and lack of options for further surgery to improve local control, amputation was discussed with patients. If the patient refused an amputation, limb-sparing therapy for recurrence according to the protocols of the interdisciplinary tumor board was initiated. Chemotherapy with doxorubicin and ifosfamide was performed only for patients with distant or lymph node metastasis.

### Statistical Analysis

The Chi-square test was used to compare categorical variables. For continuous variables, the Mann-Whitney rank-sum test was performed. For univariable analyses, Kaplan-Meier and log-rank test were performed. The primary endpoint was recurrence-free survival (RFS), measured from the date of tumor resection in our hospital to time of local recurrence, or date of the last follow-up in the absence of local recurrence. A secondary endpoint was disease-specific survival (DSS), measured from the date of the first diagnosis for the primary tumor to time of death due to sarcoma or to date of the last follow-up if no death occurred. Time periods at risk of new local recurrence and death due to sarcoma were defined in months for each patient. A *P*-value of < .05 was regarded as statistically significant. Follow-up data from patients were collected during their regular follow-up examinations according to the NCCN-Guidelines for STS (www.nccn.org/professionals/physician_gls#soft-tissue-sarcoma) in 3- to 6-month intervals, or data were collected by telephone or mail correspondence from patients about the long-term outcome.^16^ Statistical analysis was performed with SPSS (Statistical Package for the Social Sciences) software, version 23.0.

## Results

**Figure 1.**
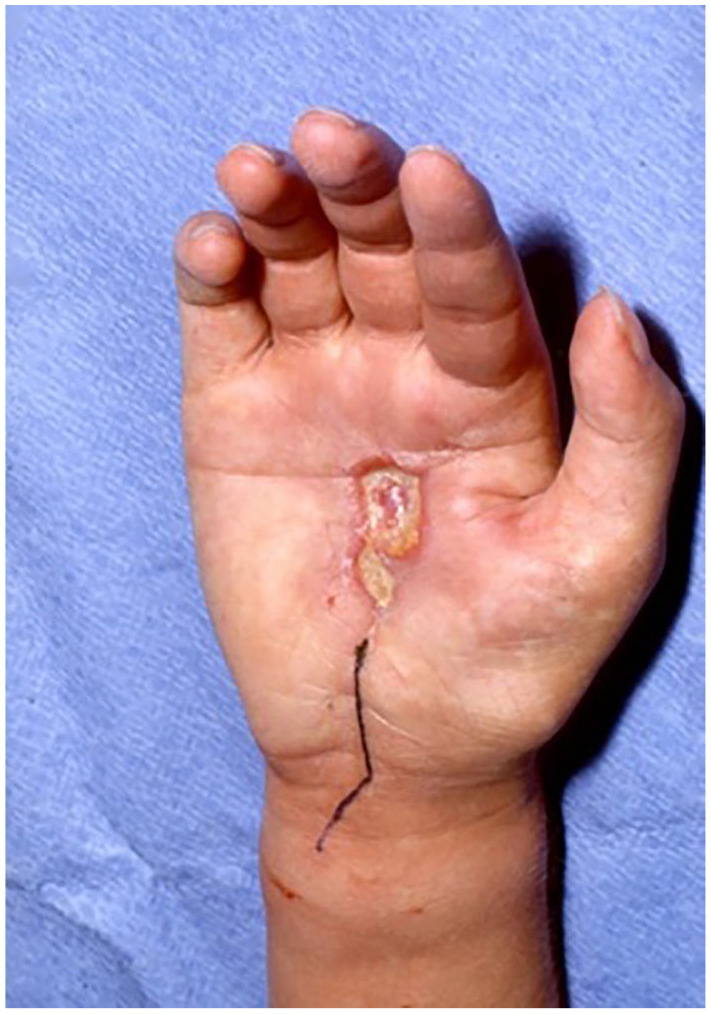
Skin lesion of the palm. Reference pathology revealed epithelioid sarcoma of the palm after the third operation in 2 years.

**Figure 2.**
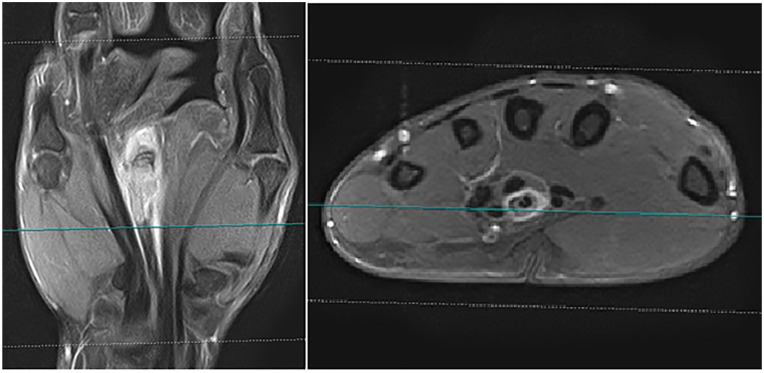
Magnetic resonance imaging with the contrast of the hand. Epithelioid sarcoma spreading along the flexor tendon of the third finger.

There were 23 patients included in the study; 13 males and 10 females. The mean age was 27 years (range: 6-76 years). Twelve patients were referred to our clinic after incomplete tumor resections in other hospitals, 8 patients with local recurrences after external primary therapy, and only 3 patients presented to our service with primary tumors. Most of the patients were affected by tumors more than 2 cm; 9 of them more than 5 cm. The palmar side of the hand, as well as the right hand, were more frequently affected by a tumor (*P* < .05). The most common site affected by a sarcoma was wrist in 47.8% of cases, followed by metacarpals and finger with 34.8% and 17.4%, respectively (Table 1). Eight patients reported painless skin lesions for more than 1 year (Figure 1), while others described tumor masses on the extremity with very slow growth. Three of the patients with skin lesions were operated on more than 3 times for false diagnoses such as Dupuytren's disease or warts. Two patients described symptoms of median nerve compression. In 20 patients, MRI revealed diffuse growth of the tumor along important anatomical structures such as tendons or nerves (Figure 2).

**Table 1.** Patient and Tumor Characteristics.

		*P*
Median age (mean and range)	26,5 (6-76)	
Sex		
Male	13 (56.5%)	>.05
Female	10 (43.5%)	
First presentation		
External insufficient tumor resection	12 (52.2%)	<.05
Local recurrences	8 (34.8%)	
Primary tumors	3 (13%)	
Tumor size		
< 2 cm	4 (17.4%)	>.05
≥ 2 cm and ≤ 5 cm	10 (43.5%)	
> 5 cm	9 (39.1%)	
Side		
Right	16 (69.6%)	<.05
Left	7 (30.4%)	
Site		
Finger	4 (17.4%)	<.05
Metacarpus	8 (34.8%)	
Wrist	11 (47.8%)	
Palmar/Dorsal		
Palmar	18 (78.3%)	<.05
Dorsal	5 (21.7%)	

Due to the small tumor size, absence of diffuse growth, and favorable location on the hand and finger, 3 patients received a wide excision without neo/adjuvant therapy. Seventeen patients were treated according to the protocols of interdisciplinary tumor boards with multimodal therapy. Five of these patients were treated with TM-ILP as a part of the multimodal therapy. Histologically, there was no response to TM-ILP in resected specimens in all patients.

Because of extensive local invasion of the tumor into important structures of the distal upper extremity, 3 major amputations were performed (Table 2).

**Table 2.** Characteristics of the First Local Treatment in Our Clinics After Confirmation of the Diagnosis.

Therapy	Patients N	Margins after tumor resection	Recurrence
Wide excision	3 (13%)	R0 (3) / R1 (0)	1
Wide excision and radiation therapy	12 (52.2%)	R0 (7) / R1 (5)	3
TM-ILP and wide excision	3 (13%)	R0 (2) / R1 (1)	2
TM-ILP, wide excision and radiation therapy	2 (8.7%)	R0 (2) / R1 (0)	0
Major amputation	3 (13%)	R0 (2) / R1 (1)	1

TM-ILP: Melphalan.

## Recurrence-Free and Disease-Specific Survival

**Figure 3.**
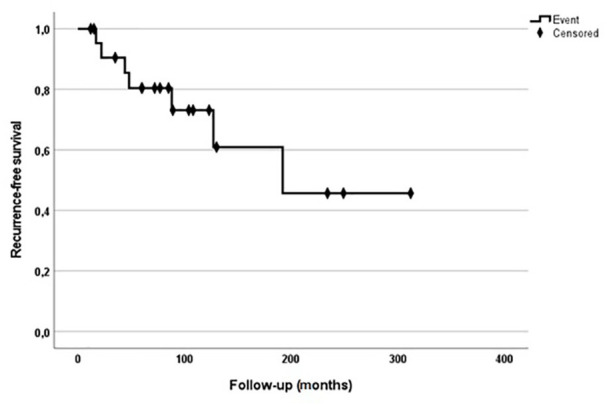
Kaplan-Meier survival curves of recurrence-free survival.

**Figure 4.**
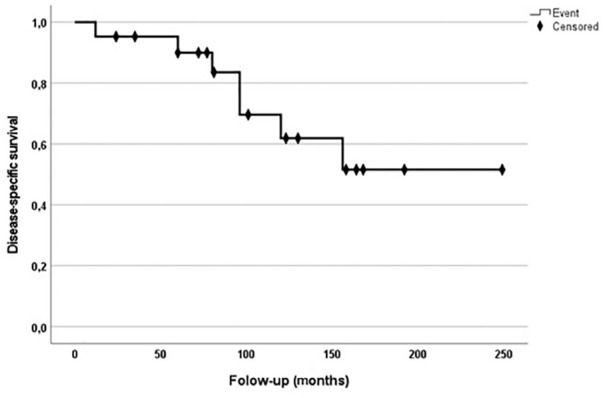
This graph shows a Kaplan-Meier disease-specific survival curve of patients with epithelioid sarcoma of the distal upper extremity.

A local recurrence was observed in 7 patients (30.4%). The median follow-up time of RFS after the first operation with the confirmed diagnosis was 85 months (range, 12-312). The 5- and 10- year RFS was 80.4% (95% CI: 68.6-76.8) and 60.9% (95% CI: 53.5-68.3), respectively (Figure 3). Three patients with recurrences received major amputation. Of 9 patients (39.1%) with disease complicated with distant metastasis, 7 of them died of disease. In all of them, tumor spread into the lung, but in 3 of them metastasis occurred also into the brain and skin of the head. The 5- and 10-year DSS was 89.9% (95% CI: 87-92.8) and 61.9% (95% CI: 56.5-67.3), respectively. Five patients (21.7%) had metastasis in regional lymph nodes. Three patients received regional lymph node dissection. Four of them died of disease soon due to distant lung and skin metastasis, and 1 patient was alive with the disease under chemotherapy at most recent follow-up (Figure 4).

## Discussion

The classic type of ES is one of the most common high-grade sarcomas of the upper extremity. According to the limited information mainly based on case reports, clinical behavior is difficult to estimate. We conducted this retrospective study to assess clinical features and long-term oncological outcome of ES of the distal upper extremity. To our knowledge, this is one of the largest patient series treated over such a long time period.

The median age of our group was 27 years, which is by far younger than in other sarcoma types.^17^ The time elapsing between the first symptoms and correct diagnosis is more than 1 year due to slow growth of the tumor and mimicry to other benign lesions, which leads to intralesional resection during the first operation.^8,18^ Timely involving of a reference pathologist is strongly recommended to avoid a delay of diagnosis.

Taking into account that ES are high-grade sarcomas and most of our patients were already surgically treated in other hospitals, multimodal therapy was predominantly used for these patients. The main goal of surgery was to achieve negative margins, which failed in some cases due to the fact that ES spreading locally through synovial spaces, along nerves, vessels, and bones.^19^ Nevertheless, 5-year RFS was estimated with 80.4%, which is not different from other sarcoma subtypes.^15,20^ But it should be noted that with local relapse, the possibility of further successful limb-sparing surgery reduces due to typical ES growth, lack of reserve of radiation therapy, and no pathological response of TM-ILP. Five patients underwent TM-ILP as a part of multimodal therapy. There was no pathological response in all resected tumors. As it is known, TM-ILP demonstrates good local control results in locoregional treatment in patients with advanced primary and recurrent extremity soft tissue sarcomas. But with a more accurate analysis of the minimal data in the literature, it can be observed that ES generally has a poor pathological response to this therapy.^21,22^

Due to the lack of (neo-) adjuvant treatment possibilities, 3 of our recurrences were treated with major amputation. These patients were admitted at first in our clinics, with recurrences after external operations. Five-year DSS, estimated at 89.9%, also does not differ from other sarcoma subtypes.^17,23^ However, the spread of the tumor in the brain and scalp makes ES unique compared to other subgroups.^24,25^

Another important feature of ES in our group is a higher risk of lymph node involvement (21.7%), consistent with the rate of 22% and 45% demonstrated in the literature.^26-29^ For comparison, only rhabdomyosarcoma and clear cell sarcoma have the same risk, while the likelihood of developing lymph node lesions by other sarcoma types is only up to 3%.^30,31^ Unfortunately, ES with lymph node metastasis is associated with a poor survival prognosis. Distant lung metastasis developed in all our patients; moreover, 4 of them died as a result of disease. There is limited research available regarding benefits of selective versus radical lymph node dissection and when each treatment should be utilized for best results, and further research is needed

The findings of this study have to be seen in light of some limitations. First, a small patient group and retrospective study design due to the rarity of these malignancies in the population. Only just over 600 cases have been reported since 1970 as case reports or in case series.^32^ In this regard, it is difficult to present an analysis that is more significant; thus, multicentral prospective study involving the national and international centers is crucial. The second limitation concerns the differences in the presentation at the time of diagnosis; most of the patients were admitted to our clinics only after recurrences or tumor resections in nonspecialized hospitals. Additionally, different localizations of the tumor on the distal upper extremity can significantly affect the therapy and oncological outcome. For example, negative margins of ES on the fingertips could be easily achieved with finger amputation, whereas, probability of positive margins after limb-sparing resection of tumor on the wrist is significantly higher due to diffuse growth typical for this type of malignancy. Therefore, this could be associated with an increased risk of local relapses.

In conclusion, the classic type of ES represents a group of high-grade sarcomas, which affect the dominantly distal upper extremity. Younger age of patients, slow tumor growth with clinical and pathological mimicry of other benign tumors makes this type of malignancy difficult to diagnose. Although long-term oncological outcome does not differ significantly from different sarcoma subtypes, high rates of lymph node metastasis and metastatic spread to the brain and skin are typical for them. Wide tumor resection as a part of multimodal therapy remains a more viable and common treatment option for patients with ES on distal extremities.
